# Regulatory Role of rno-miR-30b-5p in IL-10 and Toll-like Receptor 4 Expressions of T Lymphocytes in Experimental Autoimmune Uveitis In Vitro

**DOI:** 10.1155/2018/2574067

**Published:** 2018-10-29

**Authors:** Yuanyuan Sun, Dadong Guo, Bin Liu, Xuewei Yin, Huixia Wei, Kai Tang, Hongsheng Bi

**Affiliations:** ^1^Medical School of Ophthalmology & Optometry, Shandong University of Traditional Chinese Medicine, Jinan 250002, China; ^2^Shandong Provincial Key Laboratory of Integrated Traditional Chinese and Western Medicine for Prevention and Therapy of Ocular Diseases, Eye Institute of Shandong University of Traditional Chinese Medicine, Jinan 250002, China; ^3^Key Laboratory of Integrated Traditional Chinese and Western Medicine for Prevention and Therapy of Ocular Diseases in Universities of Shandong, Eye Institute of Shandong University of Traditional Chinese Medicine, Jinan 250002, China; ^4^Eye Institute of Shandong University of Traditional Chinese Medicine, Jinan 250002, China; ^5^Postgraduate Student of the Second Clinical Medical College, Shandong University of Traditional Chinese Medicine, Jinan 250002, China

## Abstract

Uveitis is a serious eye disease that usually damages young adult's health. MicroRNAs (miRNAs) are a class of small noncoding RNAs which regulate messenger RNA (mRNA) expression. It is predicted that rno-miR-30b-5p can regulate the expressions of interleukin-10 (IL-10) and Toll-like receptor 4 (TLR4). In this study, the regulatory role of rno-miR-30b-5p in IL-10 and TLR4 gene expressions was validated using luciferase activity assay. Further, the inflammatory manifestation of the anterior segment and pathological examination of the eye were explored in experimental autoimmune uveitis (EAU) rats. Meanwhile, the levels of rno-miR-30b-5p in eye tissues, spleen, and lymph nodes were measured using quantitative PCR (Q-PCR). IL-10 and TLR4 in spleen and lymph nodes were further separately determined by using Q-PCR and Enzyme-Linked Immunosorbent Assay (ELISA). Moreover, rno-miR-30b-5p mimic and its inhibitor were separately transfected into purified T cells, and the levels of IL-10 and TLR4 were detected using PCR, flow cytometry, and ELISA techniques. Results indicate that rno-miR-30b-5p was downregulated in spleen, lymph nodes, and eye tissues whereas the expressions of IL-10 and TLR4 at mRNA and protein levels were upregulated. The levels of IL-10 and TLR4 were negatively correlated to rno-miR-30b-5p levels. The result of in vitro cell transfection experiment indicates that IL-10 and TLR4 expressions were inhibited at mRNA and protein levels after T cells incubated with rno-miR-30b-5p mimic. However, the IL-10 and TLR4 mRNA levels were upregulated in purified T cells from spleen and lymph nodes after treatment with miR-30b-5p antagonist. In addition, there was no evident change of IL-10 and TLR4 proteins in spleen and lymph node T cells between EAU control and negative treatment groups. Flow cytometry analysis revealed that rno-miR-30b-5p mimic could reduce the number of both IL-10 and TLR4 positive cells, whereas rno-miR-30b-5p inhibitor could increase the number of IL-10 and TLR4 positive cells. Our study demonstrates that rno-miR-30b-5p influences the development of uveitis by regulating the level of IL-10 and TLR4 positive cells, thereby playing a role in the pathogenesis of uveitis.

## 1. Introduction

Uveitis is a complicated inflammatory disease of the uvea that is considered to be one of the important causes of blindness in the world [1, 2], which is usually classified according to the anatomical location of inflammation into anterior, intermediate, posterior, and panuveitis. Noninfectious uveitis includes a series of ocular inflammatory diseases that is involved in systemic immune disorders [3, 4]. T-cell–driven cellular immune responses have been confirmed to play an important role in the pathogenesis of uveitis [5–7]. Several animal models and clinical trials have been developed to address the development and pathogenesis of uveitis. Chang et al. [8] observed that Toll-like receptor (TLR) 4 and its associated lipopolysaccharide (LPS) receptor complex are widely expressed in normal human iris, ciliary body, uvea, retina, sclera, and conjunctiva. TLR 4 can be combined with other ligands of LPS to generate proinflammatory cytokines, upregulate the costimulatory factor and major histocompatibility complex (MHC), and thus activate dendritic cells, enhance their antigen-presenting ability, thereby activating the initial T cells [9]. Meanwhile, TLR2, TLR4, and other TLRs are closely related to the pathogenesis of uveitis [10, 11]. Previous study has demonstrated a higher expression of TLR4 in vivo during endotoxin-induced uveitis (EIU) in macrophages [12]. By contrast, interleukin 10 (IL-10) plays a protective role in a mouse experimental autoimmune uveoretinitis model and is upregulated in human uveitis patients [13–15]. IL-10 not only indirectly prevents antigen-specific T-cell activation but also directly inhibits T-cell expansion via inhibiting IL-2 production and thus decreases the generation of proinflammatory cytokines and chemokines, indicating its great potential therapeutical utility in treating chronic inflammatory autoimmune diseases [16]. Recent research shows that IL-10 levels were elevated in serum in EAU rats [17], while the IL-10 mRNA levels were increased in spleen and lymph nodes tissue [18]. All these findings suggest that both TLR4 and IL-10 play a critical role in the pathogenesis of uveitis.

MicroRNAs (miRNAs) are about 22 nt small noncoding RNAs which regulate the gene expression by targeting mRNAs and triggering either translation repression or RNA degradation [19]. Studies have shown that expression and regulation of miRNAs are closely associated with some diseases. At the same time, increasing evidence indicates that miRNAs play a key role in processes involved in immune system functions and pathogenesis [19]. It was found that miR-30b exerts a regulatory function in pairing innate and adaptive components of immunity; overexpression of miR-30b attenuates uptake and processing of soluble antigen ovalbumin [20]. Moreover, downregulation of miR-30b could enhance autophagy and attenuate cartilage degradation, indicating a protective role in TNF-*α*-induced apoptosis of ADTC5 cells, and the possible mechanism may be involved in the elevation of cellular survival during inflammation and therapeutic potential for inflammatory diseases [21]. Our previous study has shown that rno-miR-30b-5p expression was significantly upregulated in rat serum with experimental autoimmune uveitis (EAU), indicating that rno-miR-30b-5p may participate in the occurrence and development process of uveitis. Further bioinformatics analysis confirms that rno-miR-30b-5p can regulate the expressions of multiple target genes, including the immune-related inflammatory factors such as IL-10 and Toll-like receptors, and thus play an important role in the development and pathogenesis of uveitis [22]. Nevertheless, it is still unknown whether rno-miR-30b-5p regulates the expression of IL-10 and TLR 4 to influence the pathogenesis of uveitis.

Considering that rno-miR-30b-5p may regulate IL-10 and TLR 4 expressions and thus influence the occurrence and development of uveitis, in the present study, we verified the regulatory effect of rno-miR-30b-5p on the IL-10 and TLR 4 expressions using dual luciferase reporter system and investigated the regulatory influence of rno-miR-30b-5p on the expressions of IL-10 and TLR 4 of T lymphocytes from EAU rats in vitro. Our findings provide a valuable insight into the pathogenesis of uveitis regulated by rno-miR-30b-5p, and this finding may provide a potential molecular therapeutic target for uveitis.

## 2. Materials and Methods

### 2.1. Reagents

Interphotoreceptor retinoid-binding protein (IRBP) peptide (amino acids 1177–1191; sequence, ADG SSW EGV GVV PDV) and primers were synthesized by Shanghai Sangon Biological Engineering Technology & Services Co., Ltd. (Shanghai, China). Complete Freund's Adjuvant (CFA) and 2-mercaptoethanol were purchased from Sigma-Aldrich (St. Louis, MO, USA). Mycobacterium tuberculosis (TB, strain H37RA) was purchased from Difco (Difco Laboratories, Detroit, MI, USA). Recombinant rat IL-2, fluorescein isothiocyanate- (FITC-) conjugated TLR4 antibody, and phycoerythrin- (PE-) conjugated IL-10 antibody were purchased from Peprotech (Rocky Hill, NJ, USA), Thermo Fisher Scientific (Waltham, MA, USA), and BD Pharmingen (Mountain View, CA, USA), respectively. Phosphate buffer saline (PBS, pH 7.4, 20 mmol/L), formaldehyde, paraffin, hematoxylin, and eosin (HE) were purchased from Sinopharm Chemical Reagent Co., Ltd. (Shanghai, China). RPMI 1640 medium was purchased from Gibco; Thermo Fisher Scientific, Ltd. (Waltham, MA, USA). IL-10 (JYM0651Ra) ELISA kit was purchased from Dakewe Biotech Co., Ltd. (Beijing, China). Toll-like receptor 4 (JYM0085Ra) ELISA kit was purchased from Wuhan ColorfulGene biological technology Co., Ltd. (Wuhan, China).

### 2.2. Animals

Female Lewis rats (6–8 week-old; 160–180 g) were purchased from Beijing Vital River Laboratory Animal, Co., Ltd. (Beijing, China). Rats were housed at room temperature (25 ± 1°C) with a relative humidity of 50 ± 10%. The animal facility was under a 12 h light/dark cycle. Prior to the experiments, all rats were acclimatized to the housing room and experimental handling for 1 week. Experiments were approved by the Eye Institute of Shandong University of Traditional Chinese Medicine (2015-XK-013). Principles for the care and use of laboratory animals in research were in strict accordance with the guidelines of Care and Use of Laboratory Animals published by China National Institute of Health and the ARVO Statement for the Use of Animals in Ophthalmic and Vision Research.

### 2.3. Induction of EAU

In this study, thirty-six rats were divided into three groups: a normal control group (NC group, *n* = 9), a CFA + TB group (*n* = 9), and an IRBP + CFA + TB group (EAU group, *n* = 18). IRBP emulsification was prepared using 100 *μ*g IRBP peptide (residues 1177–1191) dissolved in PBS solution supplemented with 150 *μ*L CFA and 100 *μ*g of TB, and the final volume was 300 *μ*L. In order to induce EAU, every rat in EAU group was immunized subcutaneously with 300 *μ*L IRBP emulsification, while each rat in CFA + TB group received 300 *μ*L of emulsion, which included 150 *μ*L CFA plus 150 *μ*L PBS containing 100 *μ*g of TB. Individual in normal control group was injected with only 300 *μ*L PBS.

### 2.4. Pathological Examination

A hand-held retinal camera (Genesis-D; Kowa Co. Ltd., Aichi, Japan) was used to record the inflammatory response of the anterior segment of rats every day. After immunization for 12 days, rats were humanely euthanized, and the eyes were extracted. Subsequently, the harvested eyes were fixed in 4% formaldehyde for 24 h, embedded in paraffin blocks, and serially sectioned in the transverse plane. All sections were stained with hematoxylin-eosin (H&E) solution and were observed under a light microscope (Ti; Nikon Corporation, Tokyo, Japan). The score of eye inflammation was evaluated using the previously described criteria [23], and the severity of EAU was scored on a scale of 0 (no inflammation) to 4 (maximum inflammation).

### 2.5. Determination of rno-miR-30b-5p, IL-10, and TLR4 in EAU Rats by Quantitative PCR and Enzyme-Linked Immunosorbent Assay (ELISA)

To investigate the alterations of IL-10 and TLR4 mRNA levels, Q-PCR was performed using lymph nodes and spleen of rats on day 12 postimmunization. miRNA from eye, lymph nodes, and spleen tissues and total RNA from lymph nodes and spleen tissues were isolated using microRNA Kit (Aidlab Biotechnologies Co. Ltd., Beijing, China) according to the manufacturer's instructions. After the determinations of both purity and concentration of miRNA and total RNA, the first strand of complementary DNA (cDNA) was synthesized using the PrimeScript TMRT reagent kit (TaKaRa, Shiga, Japan). The relative primer sequences were listed in Table 1. The Q-PCR reactions were performed in a 20 *μ*L volume using Light Cycler 480 SYBR Green I Master by a real-time PCR system (Light Cycler 480 II, USA) in accordance with the manufacturer's protocols. PCR amplification was carried out starting with a denaturation step at 95°C for 10 min, followed by 45 cycles (95°C for 10 s, 55°C for 10 s, and 72°C for 20 s). Results were analyzed with Light Cycler 480 Software, version1.5.1 (Roche Applied Science) using basic relative quantification method. A melting curve was performed, and only one peak appeared to confirm the specificity of the amplification products. U6 was used as the internal reference of rno-miR-30b-5p and *β*-actin as the internal reference of rest target genes. The expressions of genes were calculated using relative quantitative measurement [24], where 2^−*Δ*ΔCt^ represented a relative expression of each target gene. Each experiment was repeated for 3 times.

For the determination of IL-10 and TLR4 protein levels, both spleen and lymph nodes were isolated and IL-10 and TLR4 levels were assayed by ELISA according to the manufacturer's instructions. The absorbance value of each well was measured at 450 nm using a multifunction microplate reader (BioTek Elx800, America). The levels of TLR4 and IL-10 in each group were calculated, and a standard curve was drawn based on the calculation, respectively.

### 2.6. Cell Culture

Briefly, spleen and lymph nodes from EAU rats were obtained on days 12 after immunization. After grinding on a 200-mesh sieve, T cells were isolated by passage through a nylon wool column, followed by a collection of the T cells using Ficoll-Hypaque density gradient centrifugation. Subsequently, cells were cultured in a 5% CO_2_ incubator at 37°C for 12 h.

### 2.7. Dual-Luciferase Reporter Gene Assay

Putative rno-miR-30b-5p binding sites in the 3'UTRs of IL-10 and TLR4 mRNAs were predicted in miRBase (http://www.mirbase.org/index.shtml). rno-miR-30b-5p mimic, rno-miR-30b-5p mimic negative control, rno-miR-30b-5p inhibitor, and rno-miR-30b-5p inhibitor negative control were purchased from Guangzhou RIBOBIO Co. Ltd. (Guangzhou, China). Either wild-type or mutated IL-10 or TLR4 3'UTR reporter plasmids (IL-10-WT, TLR4-WT, IL-10-MT, or TLR4-MT, Table 2) was blended with rno-miR-30b-5p mimic or nonspecific control microRNA (normal control) and then transfected into 293 T cells. At the indicated time point, the medium was discarded, supplemented with 35 *μ*L PBS, and 35 *μ*L luciferase substrate, and then measured the fluorescence value by a fluorescence photometer (F-7000, Hitachi, Japan). The fluorescence intensity of each reporter gene and reference gene were compared with the relative control samples.

### 2.8. Expression of IL-10 and TLR4 in T Cell after Transfection by Q-PCR and ELISA

In vitro cell transfection experiments were divided into EAU, rno-miR-30b-5p mimic, rno-miR-30b-5p mimic negative control, rno-miR-30b-5p inhibitor, and rno-miR-30b-5p inhibitor negative control groups. Cells (5 × 10^5^ cells/well, final volume: 2 ml) from either spleen or lymph nodes of EAU rats were cultured for 24 h, and then were transfected with either 50 nmol/L of mimic or 100 nmol/L of inhibitor and further cultured for 72 h using riboFECT™ CP reagent kit according to the manufacturer's protocols. To determine the alterations of IL-10 and TLR4 mRNA levels, total RNA was first isolated from cultured cells. The relative primer sequences were listed in Table 1. The Q-PCR procedures were identical to “Determination of rno-miR-30b-5p, IL-10 and TLR4 in EAU rats by quantitative PCR and Enzyme-Linked Immunosorbent Assay” section. Herein, *β*-actin was as the internal reference of target genes. Every experiment was repeated 3 times.

To measure the levels of IL-10 and TLR4 of transfected T cells with either rno-miR-30b-5p mimic or inhibitor, ELISA technique was applied. The procedures were in accordance with the manufacturer's instructions. The absorbance value of each well was measured at 450 nm using a multifunction microplate reader (BioTek Elx800, America). The levels of TLR4 and IL-10 in each group were calculated and a standard curve was drawn based on the calculation, respectively.

### 2.9. Flow Cytometry

T lymphocytes in EAU control group, mimic treatment group, mimic negative treatment group, inhibitor treatment group, and inhibitor negative treatment group were, respectively, stained with direct immunofluorescence and were measured by a flow cytometer (BD FACSVerse, NJ, USA). To analyze the transfection efficiency, cells were stimulated with leukocyte activation cocktail (BD Biosciences, USA) under a 5% CO_2_ environment at 37°C for 5 h. At the indicated time point, cells were harvested, washed with PBS twice, and then incubated with FITC-conjugated TLR4 antibody at 4°C for 30 min. For further intracellular cytokine staining, cells were incubated with either PE-conjugated IL-10 antibody isotype-matched antibody after fixation and permeabilization according to the manufacturer's instructions.

### 2.10. Statistical Analysis

Data analysis was performed using SPSS 17.0 software (SPSS, Chicago, IL, USA). Each experiment was carried out in duplicate and repeated three times. Data were represented as the mean ± standard deviation (SD). Statistical comparison of mean values was performed by one-way ANOVA followed by post hoc analysis for significance using the LSD-t multiple comparison tests. *P* < 0.05 was regarded as statistically significant.

## 3. Results

### 3.1. Pathological Changes

**Figure 1:**
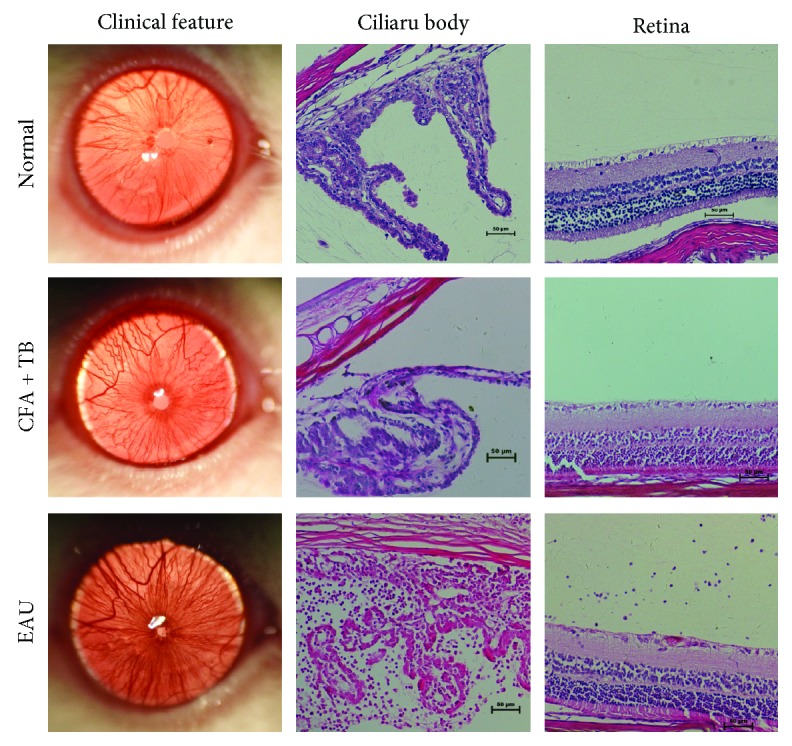
Clinical and histopathological characterizations of rat eyes and tissues from normal and EAU individuals on day 12 postimmunization. Retinal images were captured with a handheld retinal camera, and hematoxylin and eosin staining was performed on eye sections. Bar = 50 *μ*m.

On day 12 after EAU induction, we noted that compared to those of normal control group, rats in EAU group showed apparent shrunken pupil, absence of red reflex, and corneal hyperemia. The mean clinical score was 0 for NC group, 0.06 for CFA + TB group, and 3.85 for EAU group, respectively. The histopathological examination of the ciliary body and retina in EAU rats indicated that a great many inflammatory cells were infiltrated into the ciliary body and retina (Figure 1).

### 3.2. Expression of rno-miR-30b-5p in EAU Rats

**Figure 2:**
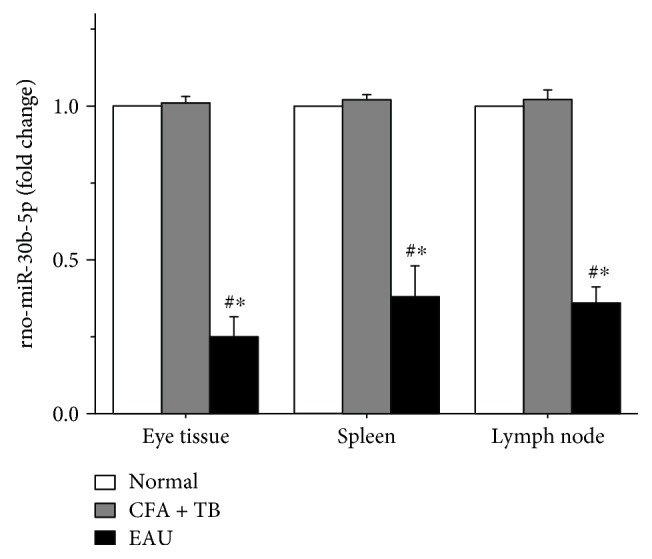
Expressions of rno-miR-30b-5p in spleen, lymph nodes, and eye tissues in EAU rats. ^#^*P* < 0.05 compared with the normal control group. ^∗^*P* < 0.05 compared with the CFA + TB group.

The results show that the levels of rno-miR-30b-5p in eye, spleen, and lymph nodes tissues in EAU rats had a marked downregulation (*P* < 0.01, Figure 2) as compared with those of NC and CFA + TB groups, respectively, indicating that rno-miR-30b-5p plays an important role in the pathogenesis of EAU. Moreover, no statistical difference was observed between the NC and CFA + TB groups (*P* > 0.05).

### 3.3. IL-10 and TLR4 mRNA and Protein Expressions in EAU Rats

**Figure 3:**
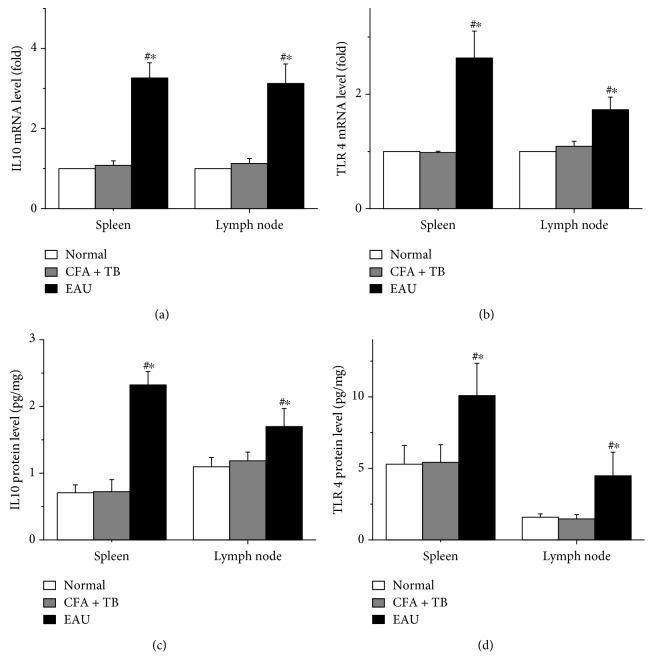
Expressions of IL-10 and TLR4 mRNA and proteins in spleen and lymph nodes in rats. (a) expressions of IL-10 mRNA; (b) expressions of TLR4 mRNA; (c) expressions of IL-10 protein; (d) expressions of TLR4 protein. ^#^*P* < 0.05 compared with the normal control group. ^∗^*P* < 0.05 compared with the CFA + TB group.

As shown in Figure 3, the expressions of IL-10 and TLR4 mRNA in spleen and lymph nodes of EAU rats increased significantly as compared with those in normal control and CFA + TB subjects, respectively, accompanied by statistical differences (*P* < 0.05, Figures 3(a) and 3(b)). ELISA tests showed that the expression trend of IL-10 and TLR4 protein was consistent with the trend of gene expression. As compared with those of NC and CFA + TB groups, the levels of IL-10 and TLR4 were statistically significant (all *P* < 0.05, Figures 3(c) and 3(d)). These results suggest that the levels of IL-10 and TLR4 mRNA are closely related to the pathogenesis of uveitis.

### 3.4. rno-miR-30b-5p Targets IL-10 and TLR4

**Figure 4:**
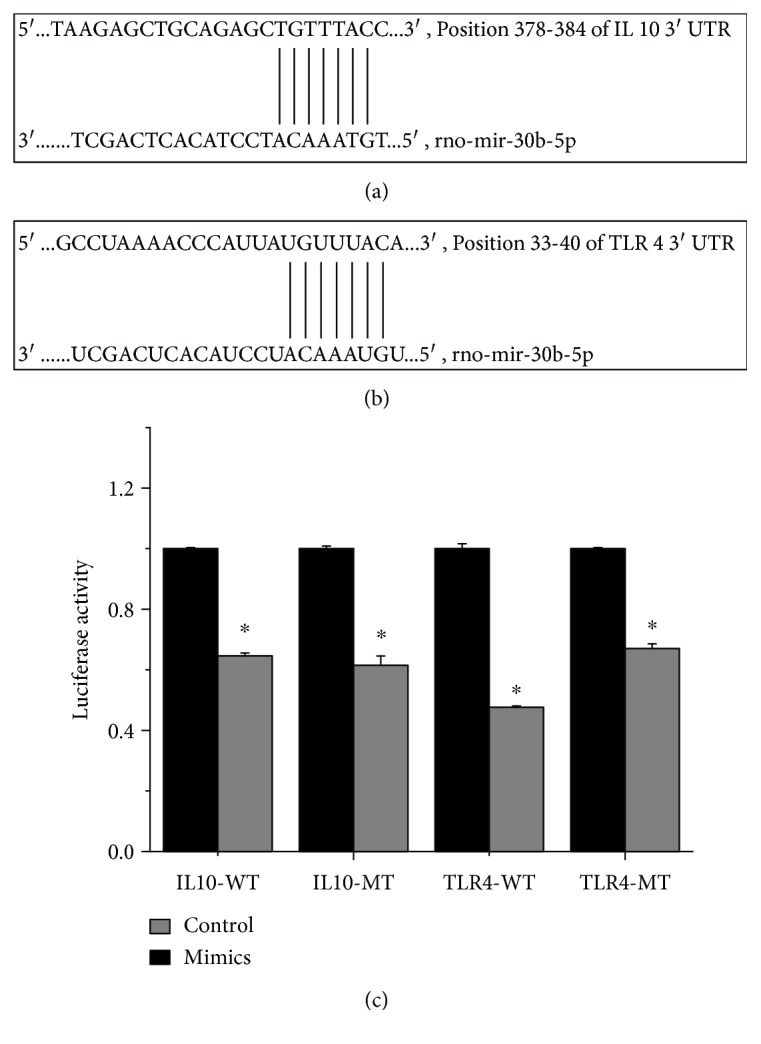
Validation of rno-miR-30b-5p targeted IL-10 and TLR4. Note: (a) sequence of 3'UTR where IL-10 mRNA bound to rno-miR-30b-5p; (b) sequence of 3'UTR where TLR4 mRNA bound to rno-miR-30b-5p; (c) dual-luciferase reporter gene assay, which indicates that rno-miR-30b-5p mimic could inhibit the luciferases activity of rno-miR-30b-5p/IL-10-WT, IL-10-MT and TLR4-WT, TLR4-MT plasmid; ^∗^*P* < 0.05; WT: wild type; MT: mutant type.

Figure 4 indicates the sequence of 3'UTR where IL-10 and TLR4 mRNA bound to rno-miR-30b-5p. It was found that rno-miR-30b-5p mimic reduced the luciferases activity of IL-10 and TLR4 wild-type reported fluorescence plasmid (*P* < 0.05) (Figure 4(c)). After the mutation of the predicted target site, the reporter fluorescence intensity in the mutant vector had also a significant downregulation (*P* < 0.05) (Figure 4(c)). These results show that rno-miR-30b-5p regulates the expressions of IL-10 and TLR4 with gene fragments of 3'UTR, but its regulatory role may have other regulatory binding sites.

### 3.5. IL-10 and TLR4 mRNA Expressions in rno-miR-30b-5p Mimic, Mimic Negative, Inhibitor, and Inhibitor Negative Groups in Spleen and Lymph Nodes

**Figure 5:**
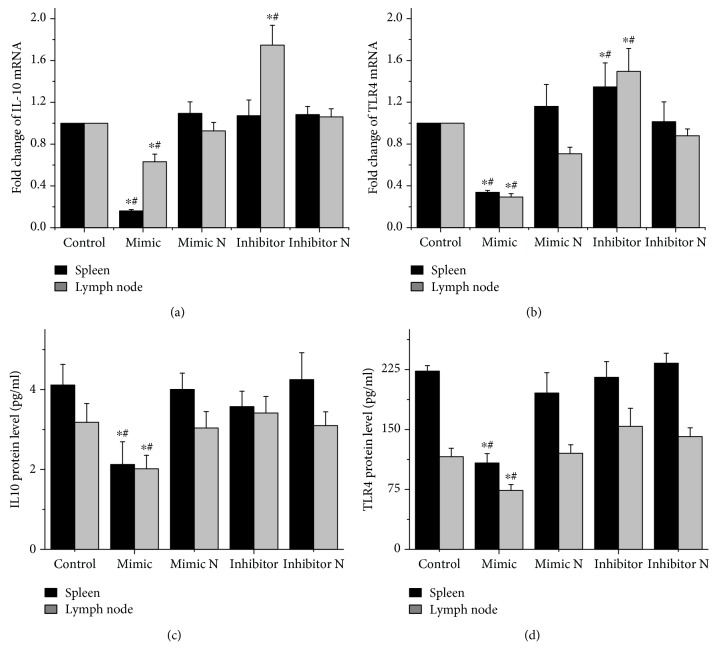
Expressions of IL-10 and TLR4 mRNA and proteins after treatment with rno-miR-30b-5p mimic, mimic negative, inhibitor, and inhibitor negative in spleen and lymph nodes. Note: (a), (b) expressions of IL-10 and TLR4 mRNA in spleen and lymph nodes; (c), (d) expressions of IL-10 and TLR4 proteins in spleen and lymph nodes. ^∗^*P* < 0.05 as compared with the corresponding EAU control, ^#^*P* < 0.05 as compared with the corresponding negative control.

The results of Q-PCR were presented in Figure 5. As it can be seen, the expressions of IL-10 and TLR4 mRNA in the mimic group in spleen and lymph nodes were significantly lower than in the EAU control group and also markedly decreased than in the mimic negative group, accompanied by a significantly statistical difference (both *P* < 0.05). At the same time, we also noted that after treatment with rno-miR-30b-5p inhibitor, the levels of IL-10 mRNA and TLR4 mRNA were apparently increased in lymph nodes as compared with either EAU control group or inhibitor negative group (both *P* < 0.05). However, the TLR4 mRNA level was markedly upregulated in rno-miR-30b-5p inhibitor-treated spleen group (*P* < 0.05), whereas the IL-10 mRNA level had no apparent alteration (*P* > 0.05). Furthermore, there was no statistical difference between the EAU control group and the negative control group.

### 3.6. IL-10 and TLR4 Protein Expressions in rno-miR-30b-5p Mimic, Mimic Negative, Inhibitor, and Inhibitor Negative Groups in Spleen and Lymph Nodes

As shown in Figure 5, the results of ELISA indicate that the expressions of IL-10 and TLR4 proteins were downregulated in T lymphocytes in spleen and lymph nodes after treatment with rno-miR-30b-5p mimic as compared with either EAU control group or mimic negative group (*P* < 0.05). However, the levels of IL-10 and TLR4 proteins had no apparent change in spleen and lymph nodes in rno-miR-30b-5p inhibitor group in comparison to either the EAU control group or inhibitor negative group (*P* > 0.05).

### 3.7. Changes of the Levels of IL-10 and TLR4 Positive T Cells in Spleen and Lymph Nodes

**Figure 6:**
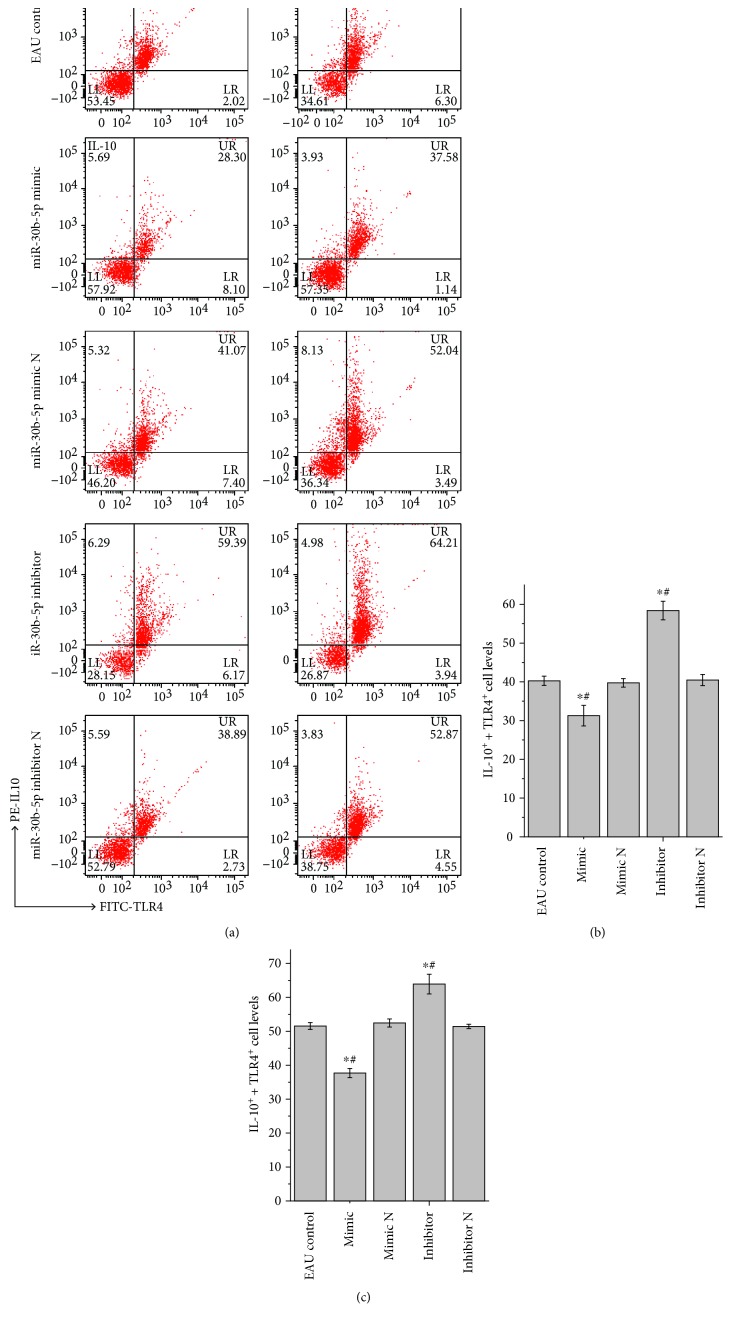
Changes in the levels of both IL-10 and TLR4 positive cells detected by flow cytometry in EAU control, rno-miR-30b-5p mimic, mimic negative, inhibitor, and inhibitor negative groups after transfection for 72 h. Note: (a) expressions of both IL-10 and TLR4 positive cells; (b) histogram analysis of IL-10 and TLR4 positive cell levels in EAU spleen after different treatments; (c) histogram analysis of IL-10 and TLR4 positive cell levels in EAU lymph node after different treatments. ^∗^*P* < 0.05 compared with the EAU control group and ^#^*P* < 0.05 compared with the relative negative control group.

As can be seen in Figure 6, compared with the relative EAU control group, the levels of both IL-10 and TLR4 positive cells were significantly decreased in spleen and lymph node groups after treatment with rno-miR-30b-5p mimic (*P* < 0.05), whereas there was no apparent change in rno-miR-30b-5p mimic N-treated groups. However, after treatment with rno-miR-30b-5p inhibitor, the levels of both IL-10 and TLR4 positive cells in spleen and lymph nodes groups were significantly increased compared with the relative EAU control group (*P* < 0.05). Nevertheless, there was no apparent change in rno-miR-30b-5p inhibitor N-treated groups. Moreover, we also noted that there was no statistical difference between either mimic negative treatment group or inhibitor negative treatment group and EAU control group.

## 4. Discussion

Uveitis is a potentially blinding group of probably multiple immune conditions predominantly occurring in the working age group easy to repeated attacks in the global scope of the blinding rate reached 10%–25% [25–27]. The development of uveitis accompanies by a series of pathological alterations involved in a large-scale of gene upregulation and/or downregulation. However, limited understanding was made for the development and pathogenesis of uveitis in gene regulation.

At present, the regulatory mechanism of miRNA has a wide impact on various physiological and pathological processes of the body, and increasing studies have confirmed the important role of miRNAs in the process of disease development. Most of the studies reveal that miR-30b plays different roles in various tumors [28]. MiR-30b-5p is one of the miR-30 family members, and its sequence is highly conserved from worms to human [29], yet the regulatory mechanism of miR-30b-5p in the development and progression of inflammatory diseases remains unclear. Currently, investigations reveal that in purified T and B lymphocytes of patients with primary Sjögren's syndrome, miR-30b-5p were enriched in the immune system that is involved in B-cell receptor signaling pathway, chemokine signaling, T-cell receptor signaling, and Fc gamma R-mediated phagocytosis [30]. Our study revealed that miR-30b-5p exhibits differential expression in EAU rats, suggesting that rno-miR-30b-5p may be closely related to the pathogenesis of uveitis. Further bioinformatics analysis shows that IL-10 and TLR4 might be the target genes of rno-miR-30b-5p [22]. In this study, we first validate that rno-miR-30b-5p can regulate the expression of IL-10 and TLR4 gene by luciferase reporter assay, and noted that even after the mutation of the predicted target site, the reporter fluorescence intensity treated with the mutant vector still showed a significant downregulation, indicating that rno-miR-30b-5p may have other binding sites, and thereby regulates the target gene expressions.

By either direct or indirect effects, a single miRNA may fine-tune the expression of thousands of genes [31]. Both bioinformatics and experiment suggest that miR-30b-5p could also downregulate the expression of Ca^2+^/calmodulin-dependent protein kinase II (CaMKII), and restoration of its function inhibits cardiac hypertrophy [32]. In addition, our previous study showed that rno-miR-30b-5p was upregulated in peripheral blood mononuclear cells in EAU rats; the predicted target genes are related to Becn1, Atg 12 and IL-10, IL21r, and TLR 4, which are involved in autophagy, immune signaling pathways [22]. It is well known that both IL-10 [33] and TLR4 [34] are involved in the development of uveitis. Therefore, we further explored the relationship between the expression levels of rno-miR-30b-5p and IL-10 and TLR4 and the development of uveitis. We observed that in spleen and lymph nodes in EAU rats, rno-miR-30b-5p was apparently down-regulated, whereas IL-10 and TLR4 were significantly up-regulated. At the same time, rno-miR-30b-5p was also found to be apparently downregulated in EAU rat eye tissues. Combined with the luciferase reporter assay result, we confirm that rno-miR-30b-5p could negatively regulate the expressions of IL-10 and TLR4, i.e., both IL-10 and TLR4 are the target genes that miR-30b-5p regulates. It would be interesting to investigate how rno-miR-30b-5p regulates IL-10 and TLR4 and thus influence the pathogenesis of uveitis. Previous studies have shown that different miRNAs have distinct mechanisms in regulating uveitis. For example, miR-155 promotes the expansion of pathogenic Th17 cells and further exacerbate the development of uveitis [35], while inhibition of miR-21 is correlated to the improvement of inflammatory BD-like symptoms through regulating cytokine expression and TLR4 [36], and hsa-miR-9 inhibits sympathetic ophthalmia through targeting TNF-*α* and nuclear factor kappa B1 [37]. In this study, we noted that rno-miR-30b-5p could efficiently negatively regulate both IL-10 and TLR4 gene expressions. The overexpression of rno-miR-30b-5p led to the significant downregulation of IL-10 and TLR4. After transfection with rno-miR-30b-5p mimic, the proportions of both IL-10 and TLR4 positive cells were significantly decreased, whereas treatment with rno-miR-30b-5p inhibitor resulted in elevated proportions compared with the relative EAU control group, and the changes of IL-10 and TLR4 levels of T cells were synchronized, though they play different roles in uveitis. It is reported that IL-10 production in macrophages is regulated by a TLR-driven transcription factor CREB-mediated mechanism that is linked to genes involved in cell metabolism [38]. Meanwhile, the activation of the TLR family has been found to be effective stimuli for inducing IL-10 production in both myeloid and B cells [39], and Okada et al. also found that infections can enhance the development of IL-10-producing B cells via TLR and thereby suppress autoimmunity [40]. Thus, we infer that TLR4 may also stimulate the production of IL-10 in T lymphocytes and thus influencing the developing processes of uveitis. Posttranscriptional gene regulation is elicited through a complex and highly interdependent network of RNA binding proteins and noncoding RNAs that form dynamic ribonucleoprotein complexes to orchestrate specific regulation of RNA transcripts throughout their lifecycle [41]. However, regarding IL-10 and TLR4, whether there are other miRNAs that can regulate the process of mRNA translation is still unclear. Therefore, more experiments are needed to be investigated.

Based on the findings that rno-miR-30b-5p targets IL-10 and TLR4 mRNA, we successfully transfected rno-miR-30b-5p mimic and inhibitor into T cells from spleen and lymph nodes, respectively. We found that the expressions of IL-10 and TLR4 mRNA and proteins were decreased in rno-miR-30b-5p mimic group. However, rno-miR-30b-5p inhibitor group has different expressions. Our results indicate that rno-miR-30b-5p can influence on the pathogenesis of uveitis by regulating the expressions of IL-10 and TLR4. However, we also noted that after treatment with rno-miR-30b-5p antagonists into T cells, the antagonist exhibited weak regulatory role in the expressions of IL-10 and TLR4. This may be attributed to the lower levels of rno-miR-30b-5p in EAU rats, and extra supplement of rno-miR-30b-5p antagonist does not play an evidently regulatory role in the expressions of both IL-10 and TLR4. Moreover, the levels of rno-miR-30b-5p differ in different tissues. Our previous study also reveals that rno-miR-30b-5p could also regulate the expressions of autophagy-related genes, such as Atg5, Atg12, and Becn1 [22]. Therefore, rno-miR-30b-5p can exert influence on the pathogenesis of uveitis through regulating various signaling pathways.

## 5. Conclusion

In summary, the present study revealed that the levels of rno-miR-30b-5p are closely associated with the pathogenesis of uveitis. Rno-miR-30b-5p is downregulated in spleen, lymph nodes, and eye tissues in rats with experimental autoimmune uveitis, regulates the levels of IL-10 and TLR4, influences both IL-10 and TLR4 positive cell proportion within cell clusters, and thus suppresses the development of uveitis. These findings provide a tantalizing hint that rno-miR-30b-5p might be a new therapeutic target for uveitis. Nevertheless, further studies are needed to detect the effect of rno-miR-30b-5p on more cell types to further support our findings.

## Figures and Tables

**Table 1:** Primer sequence of genes.

Gene	Primer sequence
rno-miR-30b-5p	RT: 5′-GTCGTATCCAGTGCGTGTCGTGGAGTCGGCAATTGCACTGGATACGACAGCTGA-3′
	GSP: 5′-GGGCTGTAAACATCCTACAC-3′
	R: 5′-TGCGTGTCGTGGAGTC-3′
U6	RT: 5′-CGCTTCACGAATTTGCGTGTCAT-3′
	GSP: 5′-CGCTTCACGAATTTGCGTGTCAT-3′
	R: 5′-GCTTCGGCAGCACATATACTAAAAT-3′
IL-10	F: 5′-TTCCATCCGGGGTGACAATAA-3′
	R: 5′-TTCTGGGCCATGGTTCTCTGC-3′
TLR4	F: 5′-GATGGCATATTTCTTGGCTTGAT-3′
	R: 5′-GGATGTCTCTATGCGATTGAAACT-3′
*β*-actin	F: 5′-CACCCGCGAGTACAACCTTC-3′
	R: 5′-CCCATACCCACCATCACACC-3′

Abbreviations: Q-PCR, quantitative polymerase chain reaction; RT, reverse transcription primer; GSP, gene specific primer; F, forward primer; R, reversed primer.

**Table 2:** miRNA target gene validation.

	Group 1	Group 2	Group 3	Group 4
Vector	3′UTR reporter (WT)	3′UTR reporter (WT)	3′UTR reporter (Mut)	3′UTR reporter (Mut)
Treatments	Normal control mimic	rno-miR-30b-5p mimic	Normal control mimic	rno-miR-30b-5p mimic

WT: wild type; Mut: mutant type.

## Data Availability

The data used to support the findings of this study are available from the corresponding author upon request.
